# Rare case of papillary fibroelastoma resection with concomitant cox-maze IV procedure

**DOI:** 10.1093/jscr/rjac371

**Published:** 2022-08-13

**Authors:** Zamaan Hooda, Ganesh Ramaprasad, Luis Cerda, Jamshed Zuberi, Mark Connolly

**Affiliations:** Department of Surgery, St. Joseph’s University Medical Center, Paterson 07503, New Jersey, USA; Department of Surgery, St. Joseph’s University Medical Center, Paterson 07503, New Jersey, USA; Department of Surgery, St. Joseph’s University Medical Center, Paterson 07503, New Jersey, USA; Department of Surgery, St. Joseph’s University Medical Center, Paterson 07503, New Jersey, USA; Department of Surgery, St. Joseph’s University Medical Center, Paterson 07503, New Jersey, USA

## Abstract

Primary cardiac tumors represent 0.1% of all cardiac tumors, making them a rare pathologic phenomenon. The second most common cardiac tumors are papillary fibroelastomas, which also represent the most common valvular tumors. This report examines a rare case of a patient that underwent resection of papillary fibroelastoma with simultaneous Cox-Maze IV procedure for treatment of atrial fibrillation. This 67-year-old male patient was initially scheduled for transcatheter ablation for treatment of rate-controlled atrial fibrillation. During a pre-procedural trans-thoracic echocardiogram, it was discovered that the patient had a moderately sized pedunculated mass on the aortic valve, suspicious of papillary fibroelastoma. Despite the patient having no history of embolic events or aortic insufficiency from the papillary fibroelastoma, the transcatheter ablation procedure was canceled. He was referred to cardiothoracic surgery for further evaluation, and it was determined that this patient was a candidate for papillary fibroelastoma resection along with Cox-Maze IV procedure for atrial fibrillation.

## INTRODUCTION

Primary cardiac tumors are rare entities with an estimated incidence of 0.1% of all cardiac tumors [1]. However, heart involvement of metastatic disease has been identified in up to 20% of cancer patients in autopsy studies [2]. Cardiac tumors may produce various signs and symptoms, such as regurgitation through interference with heart valves and heart failure, or they can also be incidental findings [3]. The majority of primary cardiac tumors are benign lesions, with the most common benign tumor being myxomas [4]. The second most common primary cardiac tumors are papillary fibroelastomas [5]. Over 80% of papillary fibroelastomas are found on heart valves, typically on the valves of the left side of the heart. These lesions are pedunculated and mobile when seen on echocardiography. Symptoms are usually secondary to embolization and include stroke, transient ischemic attack, myocardial infarction and even sudden death [6].

Unlike primary cardiac tumors, atrial fibrillation is far more prevalent and is the most common cardiac arrhythmia [7]. It is estimated that over 2 million people in the United States live with atrial fibrillation, and this figure is predicted to increase 3-to-5-fold by the year 2050 [8]. Several surgical procedures have emerged as alternatives to pharmacologic treatments for patients with atrial fibrillation. One of these surgical interventions is the Maze procedure, which involves creating multiple incisions in both the right and left atria to form scar tissue, which disrupts the electrical activity and conduction that leads to fibrillating cardiac activity seen in atrial fibrillation. The Cox-Maze procedure has recently become more popular than the traditional Maze procedure [9]. In its most advanced form, the Cox-Maze IV, energy sources such as cryotherapy and radiofrequency, are used in place of incisions to create lesions in the atria [10]. While the success of the procedure heavily depends on surgical experience and the center, reports have shown that 80–90% of patients no longer have clinical signs and symptoms associated with atrial fibrillation [11].

This report examines a rare surgical case of a patient that underwent resection of papillary fibroelastoma with concomitant Cox-Maze IV procedure for atrial fibrillation.

## CASE REPORT

**Figure 1:**
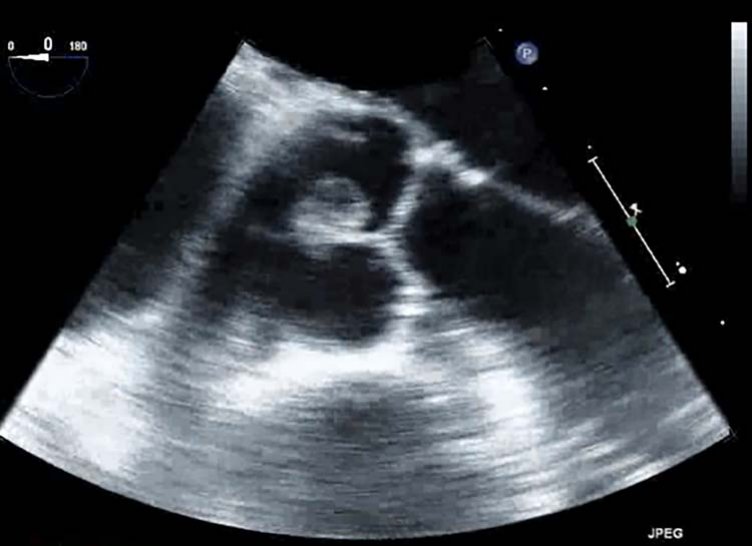
Papillary fibroelastoma seen on trans-esophageal echocardiogram as a pedunculated mass on the aortic valve measuring 16 × 8 mm.

A 67-year-old male patient with past medical history of rate-controlled persistent atrial fibrillation, hypertension, hyperlipidemia and type II diabetes mellitus was scheduled for elective transcatheter ablation for treatment of atrial fibrillation. However, during pre-procedural trans-thoracic echocardiogram, a pedunculated shimmering mass measuring 16 × 8 mm was discovered on the noncoronary cusp of the aortic valve, representing a papillary fibroelastoma (Fig. 1). This finding prompted the cancellation of the transcatheter ablation and referral to cardiothoracic surgery for further evaluation. This patient had no history of embolic events or aortic insufficiency from the papillary fibroelastoma, and the only significant cardiac finding on physical examination was atrial fibrillation; it was determined that the patient was a candidate for papillary fibroelastoma resection with concomitant Cox-Maze IV procedure for treatment of atrial fibrillation.

**Figure 2:**
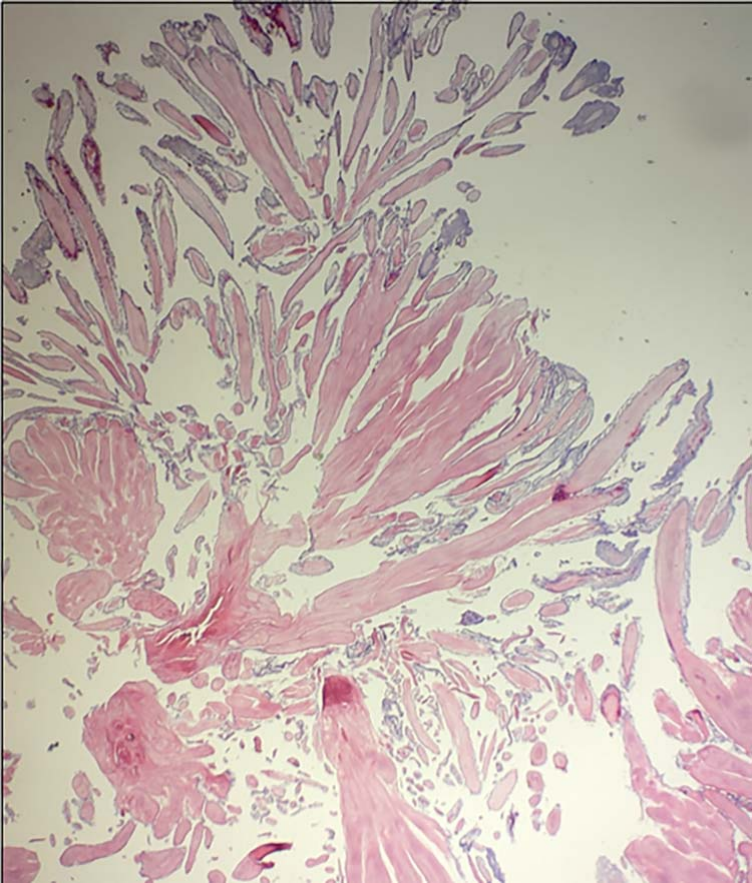
Microscopic appearance of the excised papillary fibroelastoma demonstrating avascular branches lined by endothelial cells.

The surgical procedure began with the papillary fibroelastoma resection via aortotomy. After the patient was placed on cardiopulmonary bypass and the heart was arrested, a minor aortotomy incision was made and the mass was seen attached to the noncoronary cusp of the aortic valve. The mass was shaved off and sent for further pathologic evaluation (Fig. 2), which later confirmed the mass to be a papillary fibroelastoma. After successful resection of the papillary fibroelastoma, the Cox-Maze IV procedure was performed with radiofrequency lesions created along the cuff of the left atrium, right and left pulmonary veins, posterosuperior vena cava, posteroinferior vena cava and along the atrioventricular groove on the free wall of the right atrium. Next, cryoablation lesions were made around the base of the left atrial appendage to the left superior pulmonary vein across the coronary sinus and joining the radiofrequency lesions created along the pulmonary veins and left atrium to create a box lesion around the left atrium. An additional lesion was made from the left atrial lesion to the base of the confluence between the aortic and mitral valves exteriorly and clips were placed along the base of the left atrial appendage. An intraoperative trans-esophageal echocardiogram after resection of the papillary fibroelastoma and Cox-Maze IV procedure demonstrated no residual mass on the aortic valve and no valvular insufficiency.

Three weeks following discharge after surgery, the patient was seen in the cardiothoracic surgery office for a follow-up appointment. Physical evaluation and assessment of the patient revealed that the patient had a regular rate and rhythm rather than an irregularly irregular rhythm associated with atrial fibrillation present during preoperative evaluation. An echocardiogram was performed, demonstrating a well-functioning aortic valve. The patient was also seen in clinic three months following surgery, and electrocardiogram again demonstrated normal sinus rhythm. At this time, patient no longer required amiodarone for atrial fibrillation, and is scheduled for another follow-up appointment in three months.

## DISCUSSION

This surgical report highlights the rare case of a patient undergoing resection of papillary fibroelastoma of the aortic valve along with Cox-Maze IV procedure for atrial fibrillation. Both the presence of papillary fibroelastoma and atrial fibrillation represents two unique risk factors for cerebrovascular accidents.

Cerebrovascular accidents are a notable symptom of papillary fibroelastomas, which may be due to embolization of the tumor itself or embolization of a platelet fibrin clot that develops secondary to the tumor. Therefore, prompt resection of papillary fibroelastomas upon identification is the recommended therapy [12]. When comparing patients with and without atrial fibrillation, it has been shown that atrial fibrillation is associated with a 5-fold increased risk of cerebrovascular accidents. Current pharmacologic therapy for atrial fibrillation involves rate control, rhythm control and anticoagulation [13]. Surgical therapies for atrial fibrillation include transcatheter ablation and the Cox-Maze IV procedure. The Cox-Maze IV procedure is performed at the same time as another surgical intervention that requires a median sternotomy, and is rarely performed as a stand-alone procedure. This procedure involves creating atrial lesions via radiofrequency and cryoablation, which ultimately interrupts fibrillating cardiac activity seen in atrial fibrillation [14]. Ultimately, the surgical intervention in this case reduced the likelihood of this patient having a cerebrovascular accident as it involved resecting the papillary fibroelastoma and thus far curing the atrial fibrillation.

While there is literature that demonstrates the short-term success of the Cox-Maze IV procedure in terms of treating atrial fibrillation, further studies must be conducted to assess long-term outcomes as well as studies demonstrating reduction in cerebrovascular accidents in these patients.
